# ORAL FRAILTY ASSESSMENT TOOL: INSTRUMENT DEVELOPMENT AND CROSS-SECTIONAL VALIDATION STUDY

**DOI:** 10.1093/geroni/igae098.4036

**Published:** 2024-12-31

**Authors:** Qiaoqin Wan, Yue Yang, Chengfengyi Yang

**Affiliations:** Peking University Health Science Center, Beijing, Beijing, China (People’s Republic); Peking University School and Hospital of Stomatology, Beijing, Beijing, China (People’s Republic); Peking University, Beijing, Beijing, China (People’s Republic)

## Abstract

Oral frailty is widespread in older adults, which will gradually contribute to multiple adverse health outcomes. The current assessment tools may not comprehensively assess oral frailty, thus a practical and feasible oral frailty scale is needed. The study aimed to develop an oral frailty assessment tool and evaluate its psychometric properties. The scale was developed in two stages. First, based on the Gilbert Multidimensional Conceptual Model of Oral Health, the items were generated through a literature review and Delphi expert correspondence. For psychometric analysis, content validity, structure validity, concurrent validity, convergent and discriminant validity, and internal consistency reliability of the scale were assessed. Data were collected from a convenient sample of 372 Chinese older adults, and exploratory and confirmatory factor analyses were used. The diagnostic accuracy was further conducted through receiver operating characteristic curve analysis. The newly developed scale consisted of five dimensions and 16 items. The content validity of the scale was 0.974, with 0.649 for concurrent validity, and the aggregation and differentiation validity were ideal as well. Besides, the samples were cross-validated by exploratory factor and confirmatory factor analyses. The internal consistency reached 0.865, and the retest reliability was 0.940. Meanwhile, by comparing the diagnostic accuracy with Oral Frailty Instrument-6 (OFI-6), it was found that the cutoff value was 9.5. Findings demonstrate that the Oral Frailty Assessment Tool has acceptable content validity, structure validity and reliability in measuring oral frailty. A wider range of cultures and samples are needed to refine the scale.

